# A *Kpna1*-deficient psychotropic drug-induced schizophrenia model mouse for studying gene–environment interactions

**DOI:** 10.1038/s41598-024-53237-3

**Published:** 2024-02-09

**Authors:** Hirotaka Nomiya, Koki Sakurai, Yoichi Miyamoto, Masahiro Oka, Yoshihiro Yoneda, Takatoshi Hikida, Masami Yamada

**Affiliations:** 1 Department of Cell Biology and Biochemistry, Division of Medicine, Faculty of Medical Sciences, University of Fukui, 23-3 Matsuoka Shimoaizuki, Eiheiji-cho, Yoshida-gun, Fukui, 910-1193 Japan; 2 Laboratory for Advanced Brain Functions, Institute for Protein Research, Osaka University, 3-2 Yamada-oka, Suita, Osaka 565-0871 Japan; 3 Laboratory of Nuclear Transport Dynamics, National Institutes of Biomedical Innovation, Health and Nutrition (NIBIOHN), 7-6-8 Saito-Asagi, Ibaraki, Osaka 567-0085 Japan; 4 The Research Foundation for Microbial Diseases Osaka University, Integrated Life Science Building, Osaka University, 3-1 Yamada-oka, Suita, Osaka 565-0871 Japan; 5 Department of Research and Drug Discovery, Medical Innovation Center, Kyoto University Graduate School of Medicine, 53 Shogoin-Kawahara-cho, Sakyo-ku, Kyoto, 606-8397 Japan; 6 Life Science Innovation Center, University of Fukui, 3-9-1, Bunkyo, Fukui-City, Fukui 910-8507 Japan

**Keywords:** Diseases of the nervous system, Schizophrenia, Neuroscience, Diseases, Psychiatric disorders, Schizophrenia

## Abstract

KPNA1 is a mediator of nucleocytoplasmic transport that is abundantly expressed in the mammalian brain and regulates neuronal differentiation and synaptic function. De novo mutations in *Kpna1* have been identified using genome-wide association studies in humans with schizophrenia; however, it remains unclear how KPNA1 contributes to schizophrenia pathogenesis. Recent studies have suggested a complex combination of genetic and environmental factors that are closely related to psychiatric disorders. Here, we found that subchronic administration of phencyclidine, a psychotropic drug, induced vulnerability and behavioral abnormalities consistent with the symptoms of schizophrenia in *Kpna1*-deficient mice. Microarray assessment revealed that the expression levels of dopamine d1/d2 receptors, an RNA editing enzyme, and a cytoplasmic dynein component were significantly altered in the nucleus accumbens brain region in a gene-environment (G × E) interaction-dependent manner. Our findings demonstrate that *Kpna1*-deficient mice may be useful as a G × E interaction mouse model for psychiatric disorders and for further investigation into the pathogenesis of such diseases and disorders.

## Introduction

Genome-wide association studies have identified mutations in human importin α5 (mouse importin α1; gene symbol: *Kpna1*; protein symbol: KPNA1) in psychiatric disorders^1–3^. KPNA1 is a member of the importin α family, which assists in the transport of proteins from the cytoplasm to the nucleus in eukaryotes. Importin α recognizes classical nuclear localization signals (cNLS), which are composed of basic amino acid clusters, and forms a trimeric complex with importin β that is transported into the nucleus via the nuclear pore complex. In the central nervous system (CNS), KPNA1 is the most abundantly expressed member of the importin α family. Although KPNA1 is known as an important regulator of neuronal development in mouse embryonic stem cells^4^, *Kpna1* knockout (KO) mice (also known as *Importin α5* KO mice from the human nomenclature) do not exhibit any evident anatomical abnormalities in the CNS^5,6^. However, further behavioral phenotyping of *Kpna1* KO mice has revealed psychiatric disorder-related behavioral deficits such as a prominent reduction in anxiety-like behavior and reduced acoustic startle response^7^. Thus, KPNA1 may contribute to psychiatric disorders, however its causal roles and relevant molecular and cellular mechanisms remains elusive. The emphasis in importin α research has recently shifted from nucleocytoplasmic transport to other intracellular functions^8,9^. The KPNA1 mutations identified in patients with schizophrenia are located outside of the conventional NLS recognition region, implying that KPNA1 plays a role in schizophrenia development via mechanisms other than nucleocytoplasmic transport.

Phencyclidine (PCP) is a dissociative anesthetic known to function as a N-methyl-D-aspartate receptor (NMDAR) antagonist. Similar to other NMDAR antagonists, PCP consumption results in psychoses similar to the symptoms of schizophrenia (positive symptoms, negative symptoms and cognitive dysfunction) in otherwise healthy individuals, and can exacerbate preexisting psychotic symptoms in patients^10^. Evidence from patients as well as decades of examination of PCP induced schizophrenia-like behavior (particularly hyperlocomotion) in rodents has shown that acute PCP administration induces similar changes in the brain to schizophrenia patients^11^. Furthermore, as repetitive PCP consumption in humans is known to induce psychiatric symptoms for prolonged periods^12,13^, there has been recent attention on subchronic (repetitive) administration of lower doses during neonatal and developmental stages of rodents, which can be used to model symptoms of schizophrenia better than acute administration^11,14^, and alter various brain functions such as mesolimbic dopamine release/responsivity^14–16^. The doses and timing of PCP administration to effectively model schizophrenia remain controversial, as a single paradigm of PCP treatment which completely mimics the widespread symptoms seen in schizophrenia is yet to be found. In this aspect, the 2-hit model of schizophrenia pathogenesis which assumes contribution from both genetic factors (e.g. de novo mutations) as well as environmental factors (e.g. adolescent psychosocial stress) to the pathogenesis of schizophrenia provides explanations as to why PCP administration or genetic deficits alone cannot mimic patients completely, and has led to successful approaches for developing animal models exhibiting schizophrenia-related behavioral deficits^17,18^. As explained by the 2-hit model, the interaction between genetic and environmental factors (G × E) has been suggested to have a significant impact on the pathogenesis of psychiatric disorders^19,20^, however, the underlying mechanisms remain unknown. Recently, we demonstrated G × E interaction between KPNA1 deficiency and social isolation (a rodent model of adolescent psychosocial stress) where homozygous *Kpna1* KO mice were subjected to social isolation for two weeks (age 5–8 w). In these mice we reported that social isolation further exacerbated the schizophrenia-related behavioral abnormalities caused by *Kpna1* KO^21^.

In this study, we further analyzed G × E interactions in *Kpna1* KO mice, which are used as a model for schizophrenia-like behavioral abnormalities. We performed behavioral and microarray-based gene expression analyses on *Kpna1* KO mice treated with subchronic PCP doses during adolescence. The cortico-basal ganglia-thalamus-cortical circuit is well-recognized for its significant involvement in motor control, decision-making, and cognitive activities^17^. Impairments within this circuitry are suggested as an underlying cause for a range of movement disorders and mental conditions. Consequently, we conducted gene expression analysis in the prefrontal cortex (PFc) and nucleus accumbens (NAc) areas of the brain. In addition, we used this model to investigate the role of KPNA1 in the pathophysiology of schizophrenia-related behaviors by focusing on G × E interactions. Our findings suggest that our PCP treated *Kpna1* KO mouse model may aid in furthering developing a G × E interaction model of schizophrenia.

## Methods

### Generation of *KPNA1* KO mice

In this study, we used previously reported *KPNA1* KO mice in which exons 2 and 3 of *KPNA1* have been removed (reposited to RIKEN BioResource Research Center as RBRC06031, importin α5 KO (Impα5^–/–^), using the human nomenclature of KPNA1)^6^. After backcrossing > 10 generations on a C57BL/6JJcl background with mice purchased from CLEA Japan, Inc. (Tokyo, Japan), male and female heterozygous *Kpna1* knockout mice were mated to produce Homozygous *Kpna1* knockout (KO) and wild type (WT) mice. To allow for comparison with previous studies examining the behavioral effects of *Kpna1* knockout on mice, only male mice were used in this study^7,21^.

### Housing

Mice were kept in a noise-attenuated and temperature-controlled room at 23 °C ± 2 °C on a 12 h light/dark cycle (Light: 0900–2100 Dark 2100–0900) with ad libitum access to standard mouse chow and fresh water. Mice were housed in standard cages (21 × 32 × 13 cm) together with their same sex siblings (3–6 mice per cage) for the entire duration of experiments.

### Subchronic PCP treatment

Previous studies examining G × E interaction between genetic and environmental factors has utilized a 3-week period of environmental stress administered during adolescence (age 5–8 w)^21,22^, of which the initial week (age 5w) was identified as the critical period of vulnerability to adolescent stress^23^. Furthermore, previous studies have established a 2-week subcutaneous administration of 10 mg/kg/day PCP as a chronic dosage to induce schizophrenia-related behaviors^24–26^. To target the critical period of vulnerability to environmental stress using PCP as a stress factor, we subcutaneously administered 10 mg/kg/day PCP to 5-week-old male* Kpna1* KO and WT mice for 7 consecutive days. Vehicle (Saline) was administered to the control group.

### Approval for animal experiments

All mouse experimental procedures were approved by the Animal Experimental Committee of Institute for Protein Research at Osaka University (approval ID R4-01-0) and the Animal Care and Use Committee of Kyoto University (approval ID MedKyo17071). The animal experiment plan was also approved by the Regulations for Animal Research and the Safety Management Committee for Genetic Recombination Experiments at the University of Fukui. All methods were performed in accordance with the relevant guidelines (https://arriveguidelines.org).

### Behavioral test battery

Behavioral tests were conducted after mice reached 10 weeks of age. All tests were conducted during 13:00–18:00, during the “light” phase of the light/dark cycle, to comply with facility safety regulations and experimental protocols. The behavioral test battery was designed as previously described, with minor modifications^27^. The battery consisted of an open field test (OFT), elevated plus maze test (EPM), Y-maze, inhibitory avoidance test (IA), prepulse inhibition (PPI), forced swim test (FS), and methamphetamine-induced locomotion test, performed on the same mice in the above order, with an interval of 2–5 days between tests. The order of behavioral tests in the battery were arranged so that less stressful tests were administered before more stressful tests. A total of 24 male mice were tested (Control groups: 8 WT and 5 *Kpna1* KO; PCP-treated groups: 5 WT and 6 *Kpna1* KO). All behavioral tests were conducted at the Kyoto University Medical Innovation Center.

### Open field test

Mice were placed in the center of an open field (40 cm × 40 cm × 20 cm) made of grey plexiglass and allowed to freely explore for 60 min. The total distance traveled (cm) in the first 10 min and the percentage of time spent in the center (the center 2/3 of the width and length of the field) were measured to evaluate novelty-induced locomotor activity and anxiety-like behavior. The total distance traveled (cm) over the entire 60-min session was measured to evaluate basal levels of locomotion. Video tracking of each mouse (center point) along with all other measurements were obtained using EthoVision XT 8.5 software (Noldus).

### Elevated plus maze test

The EPM test was conducted as previously described, using a plus-shaped maze with 4 arms (each arm: 30 cm × 7 cm), consisting of 2 open arms (arms without walls), 2 closed arms (arms with surrounding walls (height = 20 cm), and a center area (7 cm × 7 cm) connecting the arms^28^. Mice were placed into the maze facing a closed arm and were allowed to explore the maze for 15 min. The time spent in open and closed arms and the number of entries into open and closed arms were measured as a measure of anxiety-like behavior. Total distance travelled in the EPM was also measured. Video tracking of each mouse (center point) along with all other measurements were obtained using EthoVision XT 8.5 software (Noldus).

### Y-maze test

The Y-maze test was conducted as previously described, using a Y-shaped maze with 3 arms (length = 42 cm, wall height = 15 cm) spaced 120 ° apart^29^. Mice were placed in the center area and allowed to explore for 15 min. Video tracking of each mouse (center point) along with quantification of arm entries were obtained using EthoVision XT 8.5 software (Noldus), and the alternation rate was calculated using a previously reported R script^30^. The alternation rate (%) was calculated using the following equation:

$${\text{Alternation rate }}\left( \% \right): \, \left( {{\text{No}}.{\text{ of times all 3 arms were consecutively entered}}} \right)/\left( {{\text{Total No}}.{\text{ of arm entries}} - {2}} \right)\, \times \,{1}00.$$

### Inhibitory avoidance test

The step through inhibitory avoidance test was performed as previously described, using a two-chamber light–dark transition apparatus (Med Associates) consisting of an illuminated grey “light” chamber, a light-attenuating black “dark” chamber, and a sliding door between the two rooms^31^. The dark room was constructed from black Plexiglas, had a black fabric ceiling and a grid floor, and the floor grating was wired to an electrical power supply. On training day, the mice were placed in the light chamber with the door to the dark chamber raised open. As soon as the hind limbs of the mice entered the dark room, the door was shut, and an electric footshock (0.5 mA, 60 Hz, 1 s) was presented. After twenty-four hours, retention of aversive memory of the shock-paired dark chamber was assessed using the same procedure, except no shocks were administered. The latency of the time required to enter the dark chamber was measured on both days.

### Prepulse inhibition test and startle response

The PPI test was administered as previously described with minor alterations^21^. Briefly, the mice were placed in a chamber and exposed to 70 dB white noise background for 30 min. Each mouse received 6 pseudo-randomized sets of 7 trials (pulse-only trial, prepulse-pulse trial, and background-only trial). The total duration of each trial was 500 ms, starting with a 50 ms null period followed by a prepulse (20 ms; 74, 78, 82, 86, or 90 dB white noise pulse). The startle stimulus (40 ms; 120 dB white noise) was presented after a 100-ms delay and was followed by a 290-ms recording time. The pulse-only trial had no prepulse and consisted only of the 70 dB background noise presented for the 20 ms prepulse period. The background-only trial included neither a prepulse nor a startle stimulus and the 70 dB background were presented instead. The following formula was used to calculate the percentage (%) of PPI:

$${1}00{-}\left\{ {\left[ {\left( {{\text{mean startle amplitude on pulse-only trials}} } \right){-}\left( {{\text{mean startle amplitude on prepulse-pulse trials}} } \right)} \right]/\left( {{\text{mean startle amplitude on pulse-only trials}} } \right)} \right\}.$$

### Forced swim test

The FS test was conducted as previously described with minor modifications^21^. Each mouse was placed in a clear glass cylinder (diameter = 13.5 cm, height = 20 cm) filled to 14 cm with room temperature water (24–26 °C) and allowed to swim for 6 min. The movement of the mice in the water was recorded on video and manually analyzed at a later date using a digital stopwatch to measure the time spent immobile (sec). Immobility was defined as a lack of any movement apart from those necessary to balance or keep the head above the surface.

### Methamphetamine-induced locomotion

To habituate to intraperitoneal (i.p.) injection, mice were injected with 10 ml/kg of saline solution for 6 days. On the 7th day, mice were first given i.p. injections of saline solution and placed in an open field for 60 min, where total distance traveled (cm/60 min) was measured to assess baseline levels of locomotion. Thirty minutes after the saline trial, 1 mg/kg of methamphetamine in saline solution (10 ml/kg) was i.p. injected and mice were placed in the open field again for 60 min, where total distance traveled (cm/60 min) was measured to evaluate locomotor responses to acute methamphetamine administration. Video tracking of each mouse (center point) along with all other measurements were obtained using EthoVision XT 8.5 software (Noldus).

### Statistical analysis of behavioral experiments

All statistical analyses and data visualization of behavioral experiments were performed using Prism 8.0 (GraphPad Software, La Jolla, CA). Data are presented as the Mean ± SEM for bar graphs, with dots indicating individual data points. For box-whisker plots, data is presented as the median (center line), ± 1.5 interquartile range (box), and minimum and maximum values (whiskers). For all results other than OFT (per 5 min), IA, methamphetamine induced locomotion and PPI, the main effects of genotype and environment, and their interaction, were analyzed with a 2-way analysis of variance (ANOVA). The main effects of genotype, environment, and trial, and their interactions, for results from OFT (per 5 min), IA, methamphetamine induced locomotion, and PPI were analyzed with a 3-way repeated measures ANOVA. All ANOVA results are shown in Supplementary Table. When a significant interaction between genotype and environment was observed in the ANOVA analysis, a post hoc Tukey’s multiple comparisons test was used to analyze differences between groups differing by 1 factor.

### Dissection and sample collection for gene expression analysis

A total of 16 male mice (Control groups: 4 WT and 4 *Kpna1* KO; PCP-treated groups: 4 WT and 4 *Kpna1* KO) were used for gene expression analysis. The 10-week-old mice were anesthetized with isoflurane, cervically dislocated, and immediately dissected. Brains were removed from the skull, sliced into 1 mm thick coronal sections using a brain matrix (BrainScience Idea, Osaka, Japan), and the PFc and NAc brain regions identified using a brain atlas were removed from the coronal slices using a 1.5 mm diameter surgical punch or scalpel. Samples were immediately immersed in RNA later solution and kept overnight at 4 ºC. The RNA later solution was removed the next day, and samples were flash frozen with liquid nitrogen and kept at – 80 ºC until further use. Tissue samples were kept on ice throughout processing and extra care was taken to prevent RNA degradation.

### Gene expression analysis

RNA was extracted from the brain tissue using the ReliaPrep™ RNA Miniprep Systems (Promega, Z6111). RNA integrity numbers were then measured to confirm that the RNA was of high quality. Hybridization was performed using the Gene Kit WT PLUS Reagent Kit (Affymetrix, 902280), and microarray analysis was performed using the Clariom S mouse Assay (Applied Biosystems, 902931). The data were acquired using the GeneChip microarray analysis system GCS 3000Dx (Affymetrix). Microarray data were quality assessed and normalized using Transcriptome Analysis Console (TAC) software (Thermo Fisher); Thermo Fisher array data were normalized using robust multichip analysis (RMA). The data were filtered to include only processed signals within 90% of the overall mean. Normalized data were analyzed using Sabio Platform (https://www.subioplatform.com/ja/). DEGs between the two groups were included in subsequent analyses if the fold change factor was > 1.7-fold and the p-value was < 0.05. The p-value was calculated using Welch’s *t* test. Additionally, false discovery rate (FDR) calculations to correct for multiple testing were done according to Benjamini. Additionally, G × E interactions were investigated using two-way ANOVA on the signal intensity of each gene. Genes with a statistically significant gene-environment interaction (P < 0.05) were identified as DEGs with G × E interactions and utilized accordingly. GO and pathway analyses were performed using the above data sets, and DAVID (https://david.ncifcrf.gov), GSEA (https://www.gsea-msigdb.org/gsea/index.jsp), and Metascape (https://metascape.org/gp/index.html#/main/step1) were used^32–35^. A two-way ANOVA was performed when a dominant interaction between the genotype and environmental factor was observed. When a predominant interaction was observed between the genotype and environmental factor, post-hoc Tukey's multiple comparison test was used to analyze differences between groups differing by only one factor. Statistical analysis and data visualization were performed using GraphPad Prism 8 (GraphPad Software, San Diego, CA, USA) and Python (https://www.python.org/).

### Ethics declarations

In accordance with ethical standards and guidelines for the use of animals in research, the animal experiments conducted in this study were approved by the Animal Care and Use Committees of the Institute for Protein Research at Osaka University, Kyoto University, and the University of Fukui. All procedures involving animals were performed in compliance with relevant laws and regulations, and every effort was made to minimize any potential discomfort or distress to the animals. Prior to the commencement of the study, informed consent was obtained from the respective authorities, and proper measures were taken to ensure the humane and ethical treatment of the animals throughout the duration of the experiments. The details of the ethical approval and procedures are outlined in the Methods section of this manuscript, as required by the journal's submission guidelines (https://arriveguidelines.org).

## Results

Behavioral analyses were conducted to determine whether genetic disruption of *Kpna1* and/or the subchronic administration of PCP affect psychiatric disorder-associated behaviors. Behavioral analyses included anxiety-like behaviors (OFT and EPM), memory-related tasks (Y-maze test and IA), sensorimotor gating (PPI), depression-like behavior (FS), and hypersensitivity to psychostimulants (methamphetamine-induced locomotion test). The significant effects caused by the genotype (G; *Kpna1* KO), environmental factor (E; PCP administration), and their interaction (G × E) were assessed using analysis of variance (ANOVA) tests.

### Anxiety-like behavior

**Figure 1:**
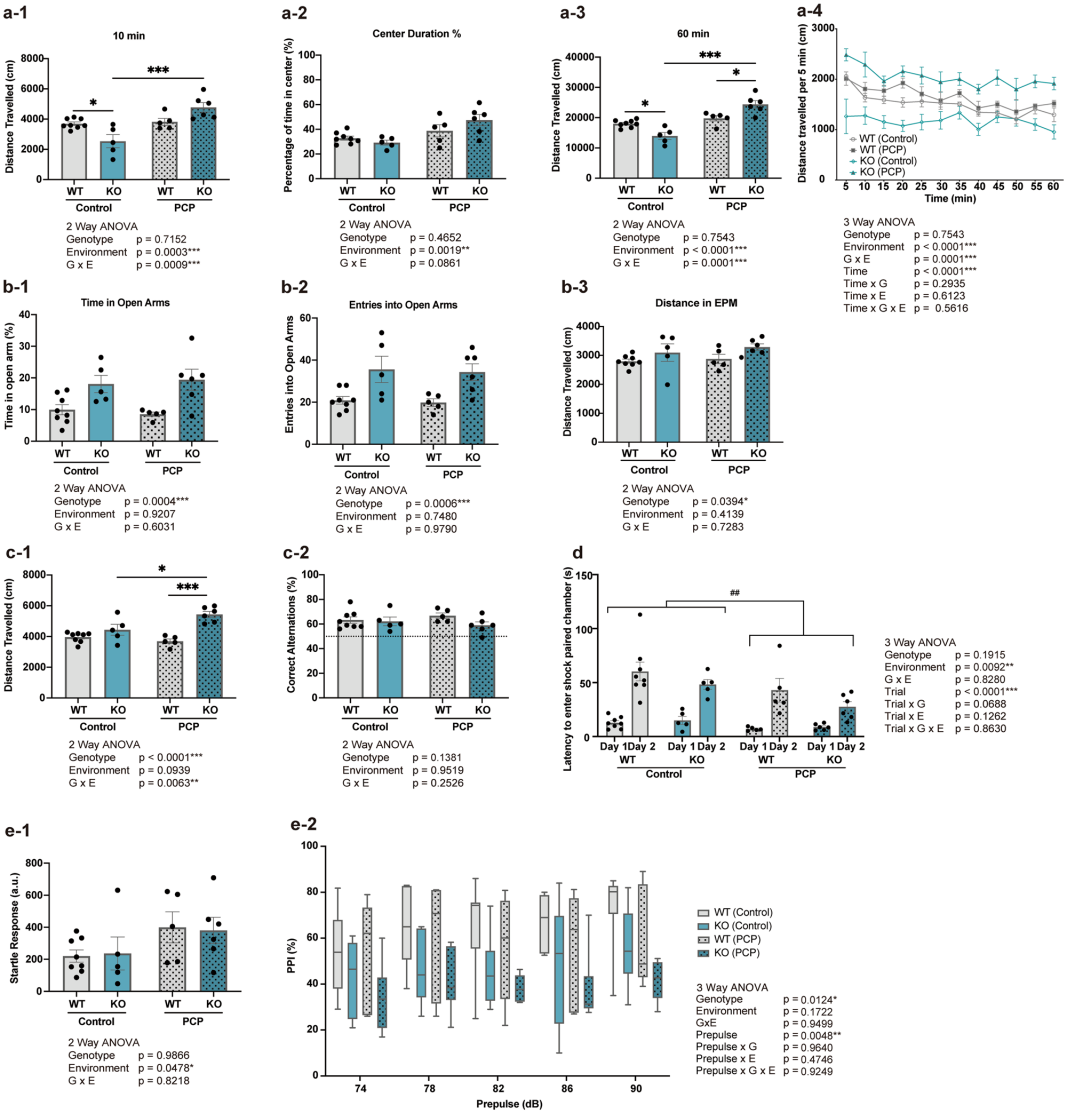
Behavioral test battery. (**a**) Assessment of anxiety-like behavior in the OFT in Control and PCP-treated WT and *Kpna1* KO mice. (**a-1**) Total distance traveled in the open field over 10 min (cm). (**a-2**) Percentage of time spent in the center of the open field over 10 min. (**a-3**) Total distance traveled in the open field over 60 min (cm). (**a-4**) Distance travelled per 5 min (cm). The data are presented as the Mean ± SEM. *p < 0.05, **p < 0.01, ***p < 0.001 Post-hoc Tukey Test. OFT, open field test; PCP, phencyclidine. (**b**) Assessment of anxiety-like behavior in the EPM in Control and PCP-treated WT and *Kpna1* KO mice. (**b-1**) Percentage of time spent in the open arms in the EPM test. (**b-2**) Total number of entries into the open arms in the EPM test. (**b-3**) Total distance travelled in the EPM test (cm). The data are presented as the Mean ± SEM. EPM; elevated plus maze test, *PCP* phencyclidine. (**c**) Assessment of short-term memory in the Y maze in Control and PCP-treated WT and *Kpna1* KO mice. (**c-1**) Total distance travelled in the Y maze test (cm). (**c-2**) Rate of correct alternations (%) in the Y-maze. The data are presented as the Mean ± SEM. *p < 0.05, ***p < 0.001 Post-hoc Tukey Test. Dotted line, 50% chance value. (**d**) Assessment of aversive learning and memory in the IA in Control and PCP-treated WT and *Kpna1* KO mice. Latency to enter the shock-paired chamber in the IA test (sec). The data are presented as the Mean ± SEM. ^##^p < 0.05 Three-way RM ANOVA. IA, inhibitory avoidance. (**e**) Assessment of sensorimotor gating in the PPI in Control and PCP-treated WT and *Kpna1* KO mice. (**e-1**) Startle response (a.u.) to a 120 dB startle pulse. The data are presented as the Mean ± SEM. (**e-2**) PPI (%) to 5 different prepulse strength levels (74, 78, 82, 86, and 90 dB). The data are presented as the median (center line), ± 1.5 interquartile range (box), minimum and maximum values (whiskers). *PPI* prepulse inhibition, *a.u*. arbitrary units.

**Figure 2:**
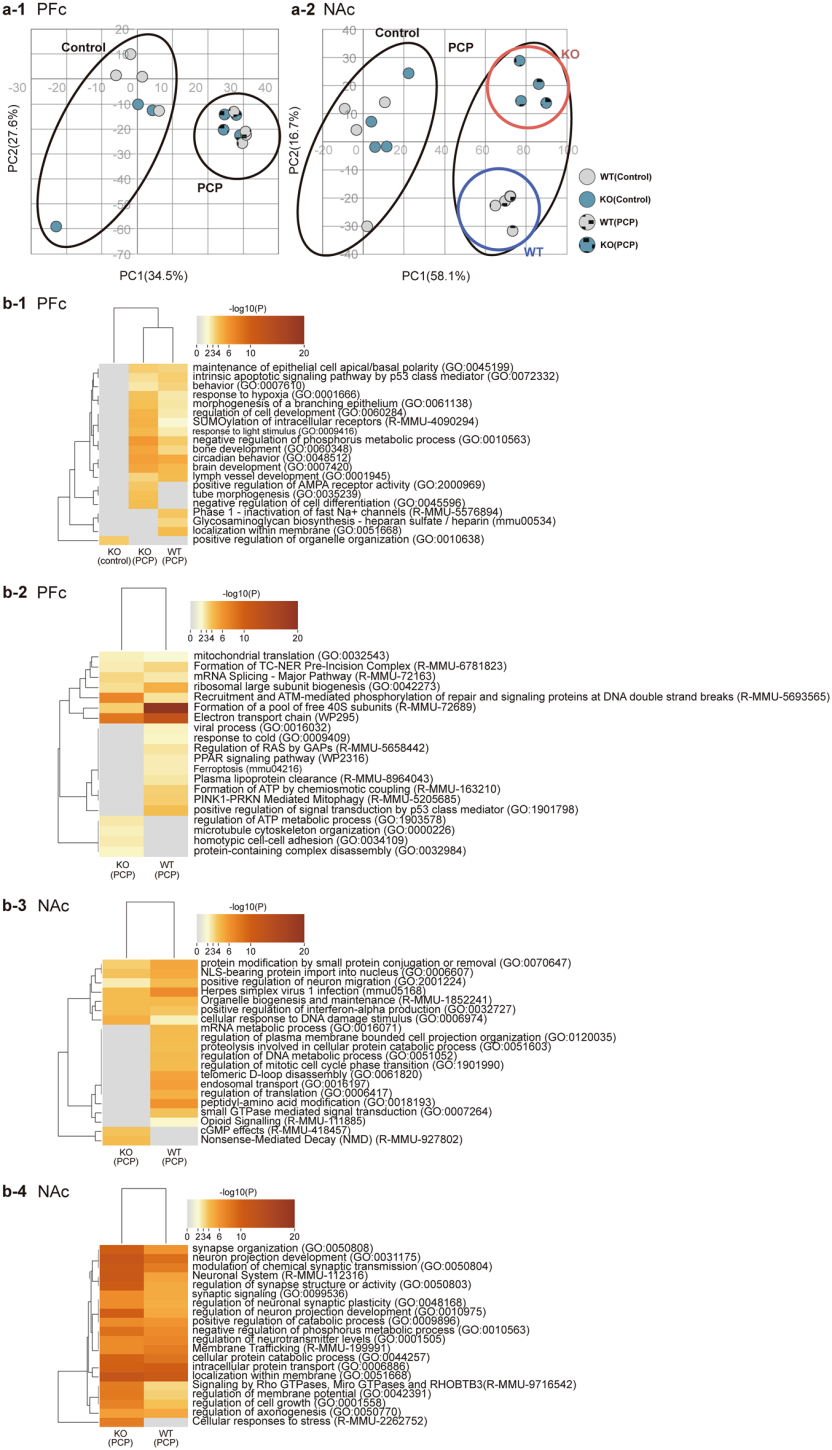
Differences in gene expression analysis in PFc and NAc. (**a**) Trends revealed using principal component analysis. (**a-1**) PCA of the DEGs in the PFc. (**a-2**) PCA of the DEGs in the NAc. Both PFc and NAc data exhibit different trends depending on the environmental factor (PCP treatment). In the NAc, the data are also dependent on the interaction between the genetic and environmental factors. Grey circles with or without dots indicate WT mice treated with saline or PCP, respectively. Blue circles with or without dots indicate Kpna1 KO mice treated with saline or PCP, respectively. (**b**) Genes with fold change greater than or less than 1.7 times and p<0.05 in Welch’s T-tests were selected as DEGs. (**b-1**) Up-regulated DEGs in the PFc. (**b-2**) Down-regulated DEGs in the PFc. (**b-3**) Up-regulated DEGs in the NAc. (**b-4**) Down-regulated DEGs in the NAc. More genes were differentially expressed following PCP-treatment in the NAc compared to the PFc. KO, Kpna1 KO; PCA, principal component analysis; DEGs, differentially expressed genes; PCP, phencyclidine; PFc, prefrontal cortex; NAc, nuclear accumbens.

**Figure 3:**
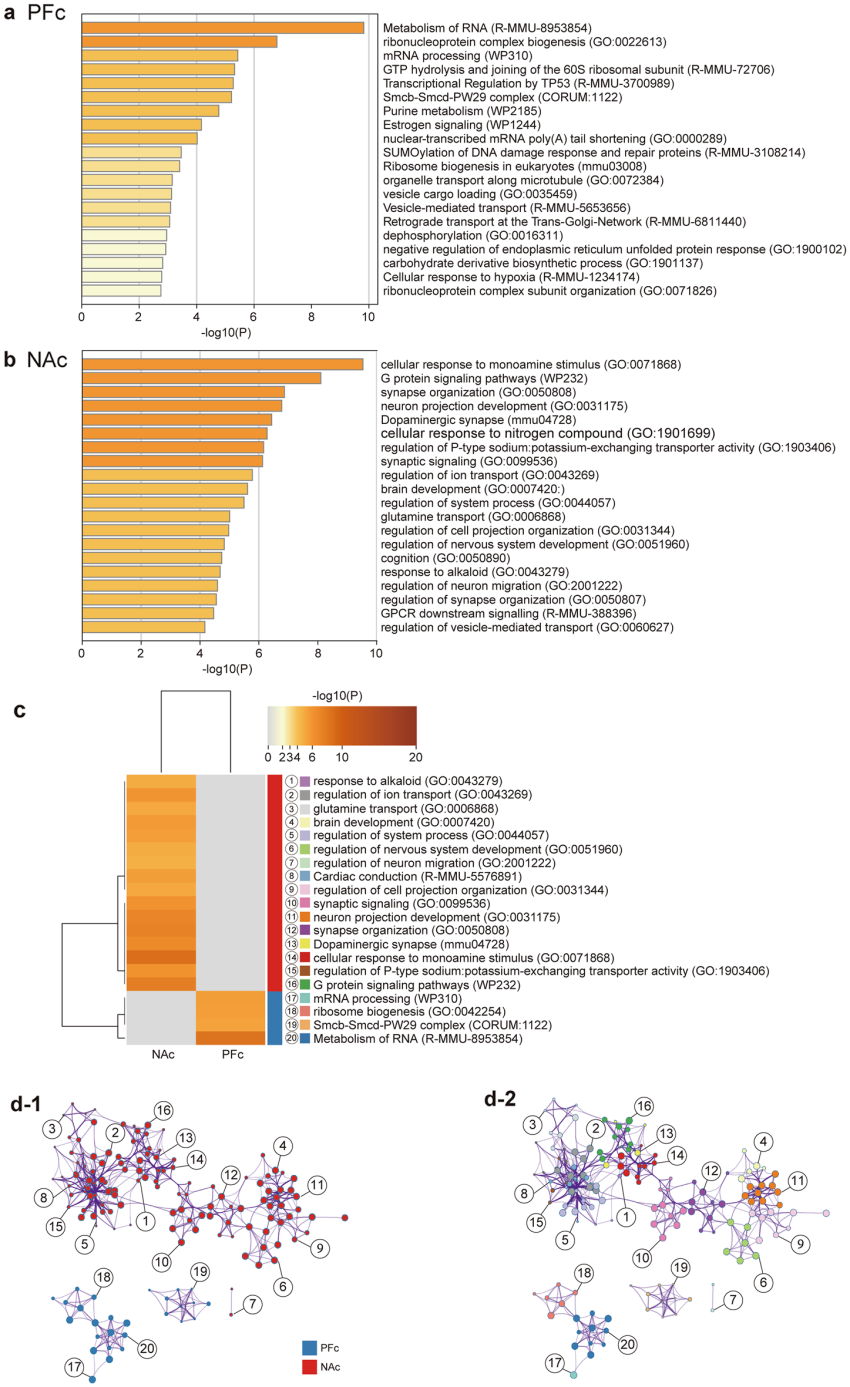
Functional analysis of DEGs affected by the G x E interaction using two-way ANOVA tests. Cluster of DEGs affected by the G x E interaction in (**a**) the PFc and (**b**) the NAc. (**c**) Clustermap colored by G x E interacting DEGs in the PFc and NAc. (**d**) Network of enriched terms colored by cluster ID, where nodes that share the same cluster ID are typically close to each other. The numbers correspond to those in (**c**) above. Each node is colored with an enriched pathway term (**d-1**). The nodes are represented by a pie chart showing their association with each input study. The color code represents the identities of gene lists (**d-2**). The PFc and NAc have very different functions for G x E interacting DEGs. Two-way ANOVA was conducted to identify factors associated with the G x E interaction. Note: DEGs that were affected by the G x E interaction in a two-way ANOVA underwent a post-hoc Tukey’s test. *DEGs* differentially expressed genes, *PFc* prefrontal cortex, *NAc* nuclear accumbens.

**Figure 4:**
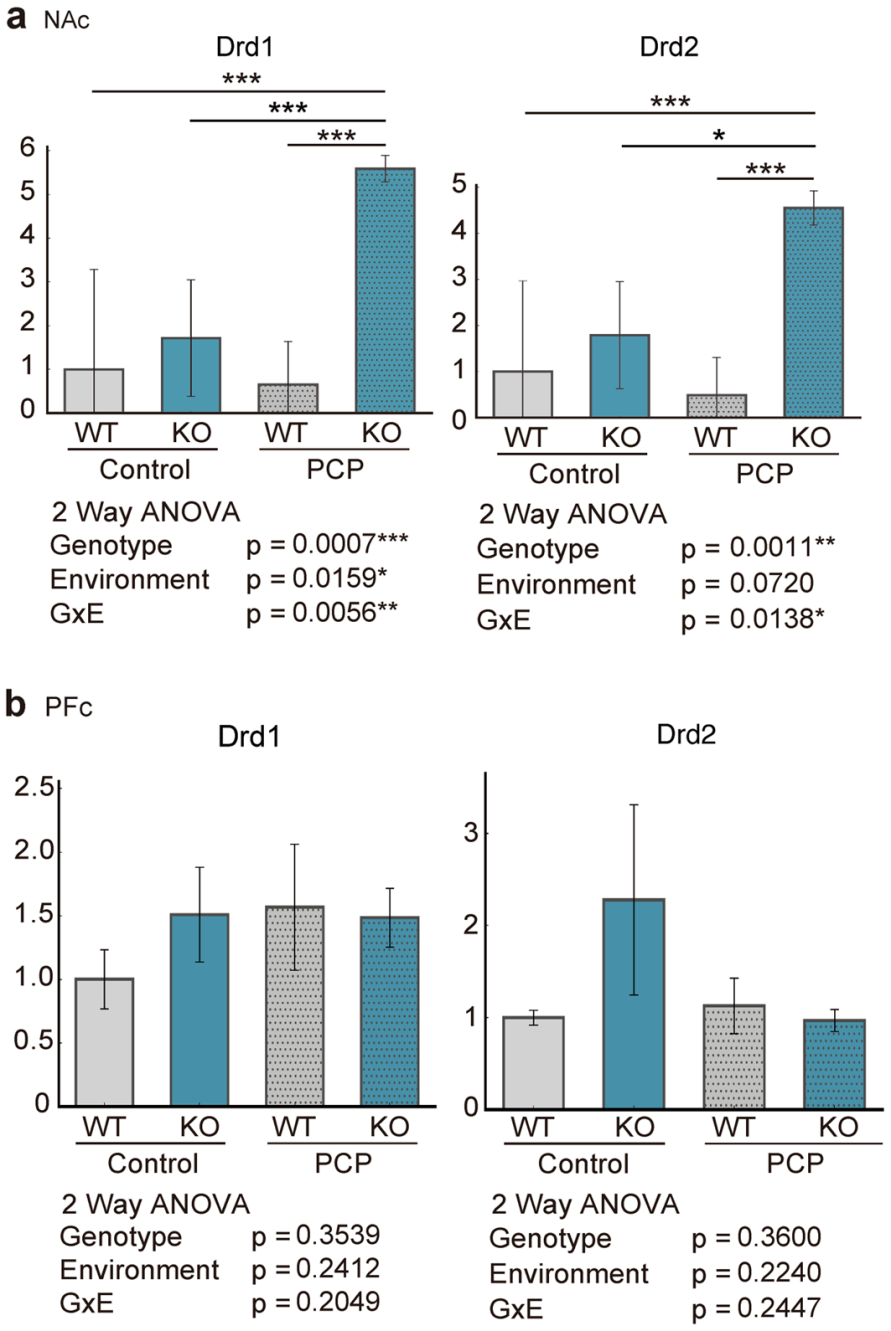
Gene expression of dopamine receptors. The WT expression level was set = 1 and the relative expression of each group is shown. Gene expression of dopamine receptors (**a**) in NAc and (**b**) in PFc. Dopamine receptor 1 (gene symbol: Drd1); Dopamine receptor 2 (gene symbol: Drd2). Two-way ANOVA tests and post-hoc Tukey tests were performed when there was a G x E interaction (p < 0.05). *p < 0.05, ***p < 0.001 Post-hoc Tukey Test.

The OFT is used to measure novelty-induced locomotion and anxiety-like responses in an unfamiliar setting^36^. Our OFT results are displayed in Fig. 1a. The total duration of the test was 60 min, of which the first 10 min were analyzed to evaluate the effects of the novel environment on the subject’s behavior (Fig. 1a-1). Anxiety-like behavior was assessed by measuring the distance travelled and amount of time spent in the center of the field during the first 10 min (Fig. 1a-2). Two-way ANOVA indicated significant main effects of environment and G × E interaction on distance traveled in the first 10 min, where PCP treatment altered behavior differently in WT and *Kpna1* KO mice. Post-hoc tests revealed a significant difference between Control WT and *Kpna1* KO groups, as well as Control *Kpna1* KO and PCP-treated *Kpna1* KO groups, where PCP treated *Kpna1* KO mice were more active in the novel environment compared to the Control *Kpna1* KO group. Moreover, a significant main effect of environment was observed on duration spent in the center of the open field, where PCP treated mice spent more time in the center of the field, indicating decreased levels of anxiety-like behavior. The basal locomotor functions of the mice were assessed by measuring the total distance traveled over the entire 60 min period (Fig. 1a-3). Two-way ANOVA analysis indicated significant effects from environment and G × E interaction. In Fig. 1a-4, the distances traveled per 5 min are shown. PCP-treated *Kpna1* KO mice exhibited increased travel distance in the unfamiliar environment for the entire 60 min. Three-way repeated measures ANOVA revealed significant effects of trial and environmental factor as well as G × E interaction. In the *Kpna1* control group, we found that the basal level of movement was significantly reduced, whereas PCP treatment increased the basal level of movement in *Kpna1* KO mice relative to PCP-treated wild-type (WT) mice. The EPM test is another widely used behavioral test to assess anxiety-like behavior^37^. In the EPM test, time spent in the open arms (%), entries into the open arms, and the total distance travelled were assessed (Fig. 1b). Two-way ANOVA revealed a significant influence on all measures of behavior assessed using the EPM test, where both *Kpna1* KO groups showed decreased levels of anxiety-like behavior compared to the WT groups.

### Memory-related tasks

The Y-maze test is used to assess short term spatial memory, where successful arm-to-arm switching into novel arms is used as a measure^38^. We found that PCP-treated *Kpna1* KO mice demonstrated more locomotion in the Y-maze (Fig. 1c-1). Two-way ANOVA analysis indicated significant main effects of genotype and G × E interaction on locomotion in the Y-maze test. Furthermore, all four treatment groups had higher levels of successful alternation compared to the 50% estimated chance value (Fig. 1c-2). Two-way ANOVA revealed that neither the genotype nor environment had significant effects on successful arm-to-arm alternation.

The IA test is used to evaluate aversive learning and memory^28,39^. In the IA test, aversion memory is assessed by measuring the latency to enter into a dark chamber paired with an aversive footshock stimulus (Fig. 1d). Three-way repeated measures ANOVA indicated that the trial (day) and environment had significant effects on the latency to enter the dark chamber.

### Sensorimotor gating

The PPI is an assessment of sensorimotor gating, a phenomenon in which a startle response elicited by a sensory stimulus is suppressed by a weak stimulus presented immediately prior to the initial stimulus. The PPI is often used as an endophenotype for schizophrenia as it is known to decrease in patients with schizophrenia^40,41^ and in numerous animal models of schizophrenia^42^. In the current study, a significant effect of environment was observed on startle response to a 120 dB sensory stimulus (Fig. 1e-1). Three-way repeated measures ANOVA indicated significant effects of the trial (prepulse strength) and genotype on startle response (Fig. 1e-2).

### Depression-like behavior

The FS test is used to assess anxiety-like behavior^43^. Two-way ANOVA analysis indicated that neither the genotype nor environment had significant effects on immobility time (Supplementary Fig. S1).

### Psychostimulant hypersensitivity test

Schizophrenia patients are known to be hypersensitive to psychostimulants, such as methamphetamine. The measurement of locomotion after methamphetamine administration in rodents has been considered a model of hypersensitive to psychostimulants found in human schizophrenia. We acutely administered methamphetamine to mice from each of the four treatment groups and measured locomotion before and after administration in an open field (Supplementary Fig. S2). Statistical analysis using a three-way repeated measures ANOVA indicated that genotype, methamphetamine administration, and their interaction (METH x G) had significant effects on locomotion. These results indicate that *Kpna1* KO mice are sensitive to methamphetamine, a phenotype comparable to human psychostimulant hypersensitivity.

Taken together, the results of the behavioral test battery indicate that the G × E interaction in *Kpna1* KO mice is characterized by decreased anxiety-like behavior (increased novelty induced locomotor activity) in the OFT, as well as increased locomotor activity in the Y-maze test. Similar to previous reports, the *Kpna1* KO genotype was associated with decreased measures of anxiety (increased open arm activity) in the EPM, increased sensitivity to a psychostimulant (methamphetamine), and decreased PPI of sensorimotor response to an acoustic startle stimulus. PCP administration alone was associated with increased center duration in the OFT, decreased aversion memory in the IA tests, and increased sensitivity to an acoustic startle stimulus.

### Trends in gene expression profiles in the prefrontal cortex and nucleus accumbens of *Kpna1* KO and PCP-administered mice.

Using a DNA microarray, we performed extensive analysis of gene expression profiles in the PFc and NAc of the four groups of mice. Principal component analysis was used to investigate the normalized data (data falling within 90% of the overall mean) and assess trends in the data for each mouse group. For both the PFc and NAc, there was a difference in gene expression profiles between the PCP-treated and saline-treated (control) groups. In addition, in the NAc, we observed a distinct difference in gene expression profiles between *Kpna1* KO mice treated with PCP and WT mice treated with PCP (Fig. 2a). We found that different brain regions expressed the genes and observed variability in expression. Overall, we identified more differentially expressed genes (DEGs) between groups in the NAc than in the PFc (Related file); for example, in the comparison of PCP-treated groups, we identified fewer DEGs in the PFc than in the NAc. These findings demonstrate that the genotype and environmental factor have distinct effects on various brain regions.

### Enrichment of gene sets in the PFc and NAc

Gene set enrichment analysis (GSEA) was performed to identify the functions of the DEGs in each brain region. Up-regulated DEGs identified in the PCP-treated *Kpna1* KO mice within the PFc were associated with behavior, circadian behavior, brain development, and positive emotions (Table 1a, Fig. 2b-1). Down-regulated DEGs within the PFc were enriched for intracellular functions, including mitochondrial translation, mRNA splicing, the electron uptake system, and the microtubule cytoskeleton (Table 1b, Fig. 2b-2). PCP-treated *Kpna1* KO mice with up-regulated DEGs in the NAc were involved in protein modification via small protein conjugation or removal, positive regulation of neuronal migration, and the cellular response to DNA (Table 1c, Fig. 2b-3). Down-regulated DEGs in the NAc were enriched for neuronal-related functions, including the development of neuronal systems and projections, synaptic organization, regulation of chemical synaptic transmission, and developmental control of synaptic plasticity (Table 1d, Fig. 2b-4). In the NAc, we observed distinct trends in the data for PCP-treated *Kpna1* KO mice and PCP-treated WT mice. PCP-treated *Kpna1* KO mice exhibited enhanced behavioral and synaptic transmission and regulation, coordination of nervous system and sensory organ development, glutamatergic synapses, and intracellular attachment (Supplementary Fig. S3a). To investigate the relationships between these terms, we constructed a network in which terms with greater than 0.30 similarity score were connected (Supplementary Fig. S3b). We observed a distinct connection between behavior, glutamate receptors, synaptic signaling, and coordination of synaptic transmission. Furthermore, we found that cell morphogenesis and intracellular adhesion are associated with head and neural system development. Enrichment scores for Kyoto encyclopedia of genes and genomes (KEGG) pathways were calculated using GSEA^32,44^. Alzheimer's disease, Parkinson's disease, Huntington's disease, axonal guidance, RNA degradation, and actin skeletal regulation were assigned enrichment scores in the PFc. We found that DEGs identified in the PFc of PCP-treated *Kpna1* KO mice and PCP-treated WT mice differed from other DEGs with regards to their positive normalized enrichment scores for Alzheimer's disease, Parkinson's disease, and Huntington's disease (Supplementary Fig. S4a). In addition, DEGs related to Parkinson's disease were also expressed among PCP-treated patients in the NAc (Supplementary Fig. S4d). This suggests that DEGs in the PCP-treated groups include important genes involved in the pathogenesis of the disease.

**Table 1:** Differences in enriched terms in each group in the PFc and NAc.

GO	Description	_LogP_KO(PCP)	_LogP_KO(saline)	_LogP_WT(PCP)
(a) Up-regulated DEGs in the PFc				
GO:0045199	Maintenance of epithelial cell apical/basal polarity	− 3.545	0.000	− 3.304
GO:0072332	Intrinsic apoptotic signaling pathway by p53 class mediator	− 3.032	0.000	− 3.538
GO:0007610	Behavior	− 2.586	0.000	− 3.302
GO:0001666	Response to hypoxia	− 3.905	0.000	− 2.654
GO:0061138	Morphogenesis of a branching epithelium	− 3.962	0.000	− 2.701
GO:0060284	Regulation of cell development	− 4.485	0.000	− 2.567
R-MMU-4090294	SUMOylation of intracellular receptors	− 4.615	0.000	− 2.054
GO:0009416	Response to light stimulus	− 4.364	0.000	− 2.522
GO:0010563	Negative regulation of phosphorus metabolic process	− 5.804	0.000	− 3.601
GO:0060348	Bone development	− 5.248	0.000	− 3.186
GO:0048512	Circadian behavior	− 5.547	0.000	− 5.076
GO:0007420	Brain development	− 5.184	0.000	− 4.231
GO:0001945	Lymph vessel development	− 3.325	0.000	− 4.139
GO:2000969	Positive regulation of AMPA receptor activity	− 3.696	0.000	0.000
GO:0035239	Tube morphogenesis	− 3.957	0.000	0.000
GO:0045596	Negative regulation of cell differentiation	− 4.001	0.000	0.000
R-MMU-5576894	Phase 1—inactivation of fast Na + channels	0.000	0.000	− 3.823
mmu00534	Glycosaminoglycan biosynthesis—heparan sulfate/heparin	0.000	0.000	− 3.218
GO:0051668	Localization within membrane	0.000	0.000	− 4.063
GO:0010638	Positive regulation of organelle organization	0.000	− 3.596	0.000

GO	Description	_LogP_KO(PCP)	_LogP_WT(PCP)	
(b) Down-regulated DEGs in the PFc				
GO:0032543	Mitochondrial translation	− 2.413	− 2.014	
R-MMU-6781823	Formation of TC-NER pre-incision complex	− 2.481	− 3.217	
R-MMU-72163	Mrna splicing—major pathway	− 3.221	− 2.633	
GO:0042273	Ribosomal large subunit biogenesis	− 3.044	− 4.694	
R-MMU-5693565	Recruitment and ATM-mediated phosphorylation of repair and signaling proteins at DNA double strand breaks	− 7.026	− 2.838	
R-MMU-72689	Formation of a pool of free 40S subunits	− 3.540	− 27.415	
WP295	Electron transport chain	− 8.109	− 12.110	
GO:0016032	Viral process	0.000	− 2.338	
GO:0009409	Response to cold	0.000	− 2.213	
R-MMU-5658442	Regulation of RAS by GAPs	0.000	− 2.763	
WP2316	PPAR signaling pathway	0.000	− 2.439	
mmu04216	Ferroptosis	0.000	− 2.437	
R-MMU-8964043	Plasma lipoprotein clearance	0.000	− 2.716	
R-MMU-163210	Formation of ATP by chemiosmotic coupling	0.000	− 3.465	
R-MMU-5205685	PINK1-PRKN Mediated Mitophagy	0.000	− 3.542	
GO:1901798	Positive regulation of signal transduction by p53 class mediator	0.000	− 4.032	
GO:1903578	Regulation of ATP metabolic process	− 2.616	0.000	
GO:0000226	Microtubule cytoskeleton organization	− 2.347	0.000	
GO:0034109	Homotypic cell–cell adhesion	− 2.558	0.000	
GO:0032984	Protein-containing complex disassembly	− 2.237	0.000	
(c) Up-regulated DEGs in the NAc				
GO:0070647	Protein modification by small protein conjugation or removal	− 3.590	− 5.175	
GO:0006607	NLS-bearing protein import into nucleus	− 3.903	− 5.063	
GO:2001224	Positive regulation of neuron migration	− 2.481	− 4.212	
mmu05168	Herpes simplex virus 1 infection	− 4.136	− 6.619	
R-MMU-1852241	Organelle biogenesis and maintenance	− 4.082	− 4.274	
GO:0032727	Positive regulation of interferon-alpha production	− 4.104	− 3.772	
GO:0006974	Cellular response to DNA damage stimulus	− 4.789	− 2.413	
GO:0016071	mRNA metabolic process	0.000	− 4.159	
GO:0120035	Regulation of plasma membrane bounded cell projection organization	0.000	− 4.082	
GO:0051603	Proteolysis involved in cellular protein catabolic process	0.000	− 4.002	
GO:0051052	Regulation of DNA metabolic process	0.000	− 4.152	
GO:1901990	Regulation of mitotic cell cycle phase transition	0.000	− 4.095	
GO:0061820	Telomeric d-loop disassembly	0.000	− 5.352	
GO:0016197	Endosomal transport	0.000	− 5.558	
GO:0006417	Regulation of translation	0.000	− 4.708	
GO:0018193	Peptidyl-amino acid modification	0.000	− 6.222	
GO:0007264	Small GTPase mediated signal transduction	0.000	− 3.882	
R-MMU-111885	Opioid signalling	0.000	− 2.145	
R-MMU-418457	cGMP effects	− 3.903	0.000	
R-MMU-927802	Nonsense-mediated decay (NMD)	− 4.101	0.000	
(d) Down-regulated DEGs in the NAc				
GO:0050808	Synapse organization	− 10.18	− 6.08	
GO:0031175	Neuron projection development	− 11.71	− 8.76	
GO:0050804	Modulation of chemical synaptic transmission	− 10.93	− 7.76	
R-MMU-112316	Neuronal system	− 11.24	− 5.38	
GO:0050803	Regulation of synapse structure or activity	− 10.47	− 4.92	
GO:0099536	Synaptic signaling	− 6.51	− 4.98	
GO:0048168	Regulation of neuronal synaptic plasticity	− 6.49	− 4.82	
GO:0010975	Regulation of neuron projection development	− 9.91	− 5.15	
GO:0009896	Positive regulation of catabolic process	− 6.70	− 5.95	
GO:0010563	Negative regulation of phosphorus metabolic process	− 8.54	− 6.58	
GO:0001505	Regulation of neurotransmitter levels	− 6.67	− 7.03	
R-MMU-199991	Membrane trafficking	− 6.66	− 7.67	
GO:0044257	Cellular protein catabolic process	− 9.67	− 8.29	
GO:0006886	Intracellular protein transport	− 9.71	− 10.12	
GO:0051668	Localization within membrane	− 12.00	− 10.79	
R-MMU-9716542	Signaling by Rho GTPases, Miro GTPases and RHOBTB3	− 7.93	− 3.27	
GO:0042391	Regulation of membrane potential	− 8.03	− 3.49	
GO:0001558	Regulation of cell growth	− 7.28	− 3.91	
GO:0050770	Regulation of axonogenesis	− 5.56	− 5.13	
R-MMU-2262752	Cellular responses to stress	− 7.70	0.00	

We identified all statistically enriched, accumulative hypergeometric p-values using each DEGs. (a) Up-regulated DEGs in the PFc. (b) Down-regulated DEGs in the PFc. (c) Up-regulated DEGs in the NAc. (d) Down-regulated DEGs in the NAc. *DEGs* differentially expressed genes, *PFc* prefrontal cortex, *NAc* nuclear accumbens.

### Differentially expressed genes affected by the G × E interaction

All genes affected by the G × E interaction were evaluated. We identified 399 G × E interacting genes in the PFc and 649 in the NAc. RNA metabolism, mRNA processing, vesicle loading, vesicle trafficking, and organelle transport along microtubules were enriched in the PFc (Fig. 3a). Cellular responses to monoamine stimulation, synapse formation, dopaminergic synapses, synaptic signaling, glutamate transport, regulation of cell projection formation, regulation of nervous system development, cognitive functions, and regulation of neuronal transport were enriched in the NAc (Fig. 3b). Additionally, there was a clear distinction between the functions of PFc- and NAc-enriched DEGs (Fig. 3c,d).

### Differentially expression of KPNAs, dopamine receptors, AMPA receptor-associated factors, and microtubule motor proteins

We next assessed the effect of KPNA1 gene disruption on the expression of other KPNAs. In the PFc, two-way ANOVA revealed that the expression of KPNA6 was affected by both the environmental factor and G × E interaction (Supplementary Fig. S5a). In the NAc, the environmental factor influenced the expression of KPNA2 and KPNA6 (Supplementary Fig. S5b).

**Figure 5:**
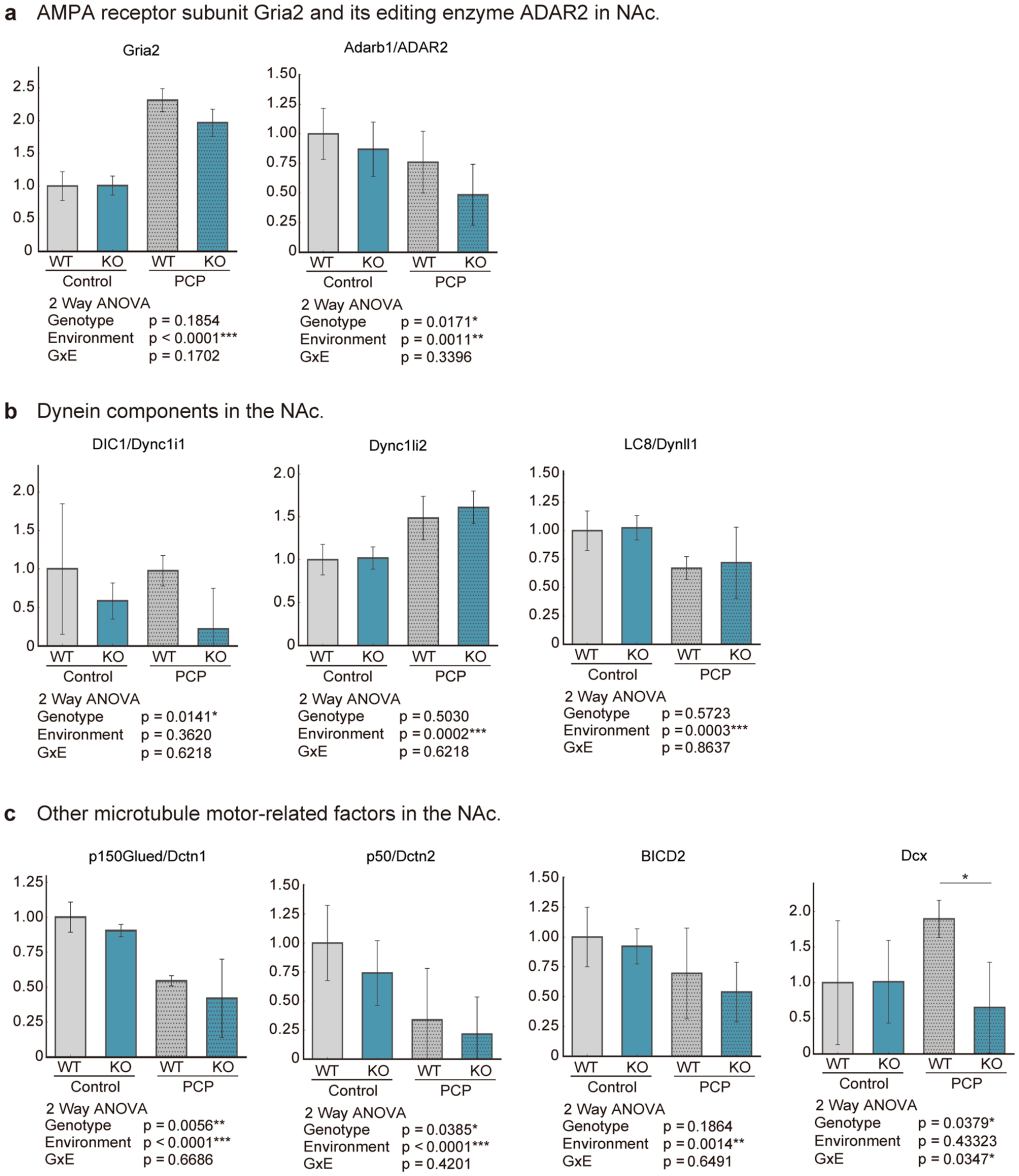
Gene expression of AMPAs and microtubule-associated factors. The WT expression level was set = 1 and the relative expression level of each group is shown. (**a**) Gene expression of AMPA receptor subunit Gria2 and its editing enzyme ADAR2 in NAc. (**b**) Gene expression of dynein components in the NAc. (**c**) Gene expression of dynactin 1/2, BICD2 and doublecortin in the NAc. Two-way ANOVA and post-hoc Tukey tests were performed when there was a G x E interaction (p < 0.05). *p < 0.05, Post-hoc Tukey Test.

Prior analyses have demonstrated that DEGs associated with behavior are enriched in the NAc. We investigated the expression of candidate genes in PCP-treated *Kpna1* KO mice relative to that in WT (saline-treated) mice. We found that PCP-treated *Kpna1* KO mice exhibited characteristic changes in dopamine receptor gene expression (Fig. 4). Two-way ANOVA was performed on dopamine receptor D1 (Drd1) and Drd2 expression and revealed that the G × E interaction influenced their expression in the NAc. Next, due to the observed variation in functional factors involved in RNA editing, we examined the expression of RNA-editing enzymes. Specifically, we focused on the RNA-editing enzyme adenosine deaminases acting on RNA (ADAR), which edits subunits of α-amino-3-hydroxy-5-methyl-4-isoxazolepropionic acid (AMPA)-type glutamate receptors, and AMPA receptor subunits (Fig. 5a). Two-way ANOVA revealed that the environmental factor was the main factor influencing the expression of glutamate ionotropic receptor AMPA-type subunits 2 (Gria2). In addition, two-way ANOVA revealed that the genotype and environmental factor affected Adarb1 expression (protein symbol: ADAR2). The expression of Gria2 increased and the expression of its editing enzyme, ADAR2, decreased in the NAc of PCP-treated KPNA1 KO mice.

The expression of genes related to microtubule motor proteins, such as dynein, dynactin, and doublecortin, was also analyzed (Fig. 5b,c). For cytoplasmic dynein, the gene encoding the intermediate chain, Dync1i1/Dc1i1/DIC1 (protein name: dynein cytoplasmic 1 intermediate chain 1), was primarily affected by the genotype. The environmental factor was found to be the main influencer of the expression of the light intermediate chains Dync1li2/Dncli2 (protein name: dynein cytoplasmic 1 light intermediate chain 2), light chains Dynll1/Dlc1/LC8 (protein name: dynein light chain LC8-type 1). Two-way ANOVA showed no main effect in G × E, but PCP-treated *KPNA1* KO mice had markedly decreased expression of DIC1 and increased expression of Dync1li2. In the NAc, PCP-treated *KPNA1* KO mice may have altered proportions of dynein components. We also analyzed other factors related to microtubule-based transport, such as dynactin and doublecortin (Fig. 5c). Two-way ANOVA revealed that the genotype and environmental factor affected the expression of Dctn1/p150Glued, the gene encoding dynactin subunit 1 (DCTN1/p150), which is a major component of dynactin. Furthermore, the genotype and environmental factor were the primary factors affecting Dctn2/p50, dynactin subunit 2 (DCTN2/p50). For Bicaudal D2 (BICD2), which is a dynein-dynactin interacting factor, two-way ANOVA revealed a primary effect of environment. Expression of doublecortin (Dcx), which encodes the microtubule-related protein DCX, was influenced by the genotype and G × E interaction. All of these microtubule-associated factors were suppressed in the NAc of PCP-treated *KPNA1* KO mice.

In summary, the expression levels of dopamine receptors, AMPA receptor-associated factors, and microtubule motor proteins were remarkably altered in PCP-treated *Kpna1* KO mice.

## Discussion

In this study, we created a G × E interaction model for schizophrenia-like behavior using *Kpna1* deficiency and adolescent PCP treatment as genetic and environmental factors, respectively. We examined the effects of *Kpna1* KO and subchronic PCP administration by subjecting the mice to a behavioral test battery and conducting comprehensive genetic analysis using a microarray. Our G × E model mice exhibited schizophrenia-related behavioral abnormalities, suggesting their possible utility as a new mouse model for schizophrenia. We also demonstrated that KPNA1 modifies PCP sensitivity to a drug stress in the NAc. Our genetic analysis suggested that behavioral abnormalities may be associated with the increased expression of both Drd1 and Drd2 dopamine receptors in the NAc.

In our behavioral test battery, *Kpna1* deficient groups exhibited a significant reduction in anxiety-like behavior in the EPM, a significant reduction in PPI levels, as well as an increased sensitivity to methamphetamine induced hyperlocomotion. This result is consistent with previous studies that have reported decreased levels of anxiety-like behavior and/or PPI levels in *Kpna1* KO mice^7,21^. Such findings provide further evidence that KPNA1 induces behavioral modifications associated with psychiatric symptoms. In addition, significant main effects of our subchronic PCP administration paradigm were observed on locomotion in the OFT (10 min, 60 min), decreased anxiety-like behavior in the OFT, decreased aversion learning in the IA, and an increased acoustic startle response. Most importantly, a significant G × E interaction was seen in subchronic PCP-treated *Kpna1* KO mice, which exhibited a significant increase in locomotion in the OFT (10 min, 60 min) and Y-maze, a measure which has been established as a model for the positive symptoms of schizophrenia^11^, suggesting that this subchronic PCP-treated *Kpna1* KO mice can be used as a model for assessing schizophrenia-related behavioral abnormalities.

PCP is a drug that causes schizophrenia-like behavioral abnormalities^10,45^ and its administration has been used as a model for schizophrenia^10,11,14^. In general, administration of PCP results in an increase in locomotor activity, a decrease in PPI, and a decrease in cognitive functions. Interestingly, we observed a significant increase in locomotion after subchronic PCP administration to *Kpna1* KO mice, despite the fact that the PCP paradigm alone was not sufficient to induce a significant increase in locomotion; this suggests a synergistic effect of PCP treatment (the environmental factor) with *Kpna1* KO (the genotype). Indeed, such results are in line with the 2-hit model of schizophrenia pathogenesis, which assumes contribution from G × E interactions between genetic and environmental factors. Subchronic phencyclidine treatment induced more behavioral abnormalities in *Kpna1* knockout mice. However, the drug effects appeared mild compared to anomalies in mice without drug treatment. De novo mutations in *Kpna1* in patients with schizophrenia are heterozygous and may not result in completely non-functional mutations. It is pertinent to assess gene-environment interactions in *Kpna1* heterozygous knockout mice or those with hypofunctional alleles. A similar behavioral analysis in *Kpna1* heterozygous mice showed no changes in responses compared to wild-type mice, even after subacute phencyclidine administration. Administering phencyclidine to *Kpna1* knockout mice during an equivalent adolescence stage in humans revealed reduced anxiety-like behavior and a substantial increase in motor activity. These results emphasize the significance of gene-environment interactions involving Kpna1.

Dopamine has been hypothesized to play a role in the pathogenesis of schizophrenia. This hypothesis is supported by the efficacy of antipsychotic drugs that inhibit the D2 dopamine receptor in treating schizophrenic patients and by the fact that psychiatric symptoms produced by CNS stimulants affect the dopamine system. These findings indicate that alterations in the dopamine system, such as dopamine overproduction and release, dopamine receptor overstimulation, and abnormal sensitivity, contribute to the etiology of schizophrenia^46^. Increased dopamine levels and Drd2 density have been reported in the striatum^47,48^, and subsequent human imaging studies have reported modifications in the striatal dopamine system^49^. These findings are consistent with the significant increase in the expression of Drd2 in the NAc of PCP-treated *Kpna1* KO mice and the absence of alterations in the PFc. Thus, we hypothesize that the behavioral abnormalities reminiscent of schizophrenia observed in our mouse model are related to changes in Drd2 expression.

Although a link between elevated Drd2 expression and schizophrenia-like behavioral abnormalities has been suggested, it remains to be seen whether there is a link between the other dopamine receptor Drd1 and behavior. We assumed that Drd1 may play a role in the absence of behavioral abnormalities in our mouse model. Memory impairment is a symptom of schizophrenia that can be induced by PCP administration^10^ and has been replicated in a number of schizophrenia mouse models^50,51^. However, behavioral analysis of our G × E model did not reveal any obvious memory deficits. Surprisingly, memory impairment-related genes were enriched in the NAc of PCP-treated *Kpna1* KO mice. The absence of memory impairment in our G × E model may be attributable to the difference in the environmental factors administered to the mice. Several studies have indicated that Drd1 agonists are effective in treating memory impairment^52,53^. In our study, a significant increase in Drd1 expression was observed in the NAc of PCP-treated *Kpna1* KO mice. This increased Drd1 expression may mitigate the memory impairment caused by PCP treatment. However, these putative effects of Drd1 on cognitive function remain controversial^54^. Notably, it has been reported that Drd1 has antidepressant effects, and the mice in our study did not exhibit depression-like behavior in the FS test; thus, it is possible that Drd1 receptors are associated with the FS outcomes in our mouse model^55,56^. Alteration in Drd1 expression may account for the absence of memory impairment and depressive symptoms in our G × E model mice, as observed in other models of schizophrenia.

According to previous reports, patients with schizophrenia have a dysfunctional AMPA receptor^57–59^; however, the mechanism by which AMPA receptors contribute to the pathogenesis of schizophrenia is not yet understood. In the NAc, we observed the altered expression of ADAR2, which is editing enzyme of the AMPA receptor subunit, GRIA2/GluR2^60^. It has been reported that the Q/R site of GRIA2 is 100% edited under normal conditions^61^, while unedited GRIA2 Q/R sites increase intracellular Ca^2+^ influx. Unedited AMPA has also been reported in the striatum of brains from schizophrenic patients^62^. AMPA receptors are composed of GRIA/GluR1-4 subunits and are one of the receptors for the neurotransmitter, glutamate. In our mouse model, ADAR2 expression was reduced, whereas GRIA2 expression was elevated in the NAc, indicating a rise in unmodified GRIA2 and leading to an increase in Ca^2+^ influx. Our gene analysis suggests that AMPA receptor dysfunction due to abnormal RNA editing may be involved in schizophrenia pathogenesis.

Our genetic analysis revealed the enrichment of microtubule-based transport in the NAc of PCP-treated *Kpna1* KO mice. The microtubule-associated motor protein, kinesin, has been reported to be associated with schizophrenia^63,64^. It has been reported that the role of dynein in axonal transport is important for brain development^65^. In this study, we also observed altered expression of the dynein capturing factor, dynactin, and a microtubule-related protein, DCX. DCX is also essential for normal brain development and is involved in neurogenesis and neural migration^66^. Numerous schizophrenia-related genes are involved in neuronal differentiation and migration, and abnormal neurogenesis has been linked to DCX expression in schizophrenia patients^67,68^. It is possible that microtubule transport is involved in the pathogenesis of schizophrenia^69,70^.

In our mouse model, fewer DEGs were enriched in the PFc compared to the NAc; this is consistent with a similar trend observed in the PFc of postmortem brains from schizophrenic patients^71^. In the neural circuitry of the brain, the NAc receives projections from the PFc via glutamatergic neurons. The NAc is a site of dopaminergic modulation of neurotransmission and may be prone to dysregulation of genes characteristic of schizophrenia^72^. It has also been reported that chronic dysregulation of the NAc triggers altered gene expression in the PFc^73^. If our model reflects the early onset of schizophrenia, then it is possible that the pathogenesis of schizophrenia originates in the NAc. The enriched function of G × E interacting genes is clearly different in the PFc and NAc, and enrichment in the NAc is associated with behavioral abnormalities, suggesting that increased vulnerability to PCP in the NAc following *Kpna1* KO is involved in the development of schizophrenia.

KPNA1 dysfunction in the NAc contributes to schizophrenia development. In our mouse model, we observed the elevated expression of KPNA6 (importin α7 in humans and importin α6 in mice) in the NAc. KPNAs are divided into three families based on their amino acid sequences homology and subtypes within the same subfamily exhibit similar substrate specificity. KPNA1 and KPNA6 belong to the same subfamily, indicating that KPNA6 may rescue the loss of KPNA1. Because numerous substrates are recognized by multiple KPNAs, the genetic deletion of any one KPNA typically has little effect^74^. This indicates that the observed expression changes and behavioral abnormalities may involve functions other than the previously identified nucleocytoplasmic mass transport of KPNA1. Since KPNA1 has substrate specificity for the transcription factor STAT1, we cannot rule out the possibility that STAT1-related functions are involved^65,75^. Future research is required to determine whether the cytoplasmic function of KPNA1 is involved in microtubule trafficking.

Behavioral abnormalities and altered gene expression in mice do not always necessarily correspond with schizophrenia in humans. Due to the diversity of environmental factors and drug treatment interventions, additional verification is needed to determine whether the present results are applicable to patients with schizophrenia. However, we propose that our model is valid for evaluating the role of genetic and environmental factors as well as their interactions in the schizophrenia pathogenesis.

In all, we conducted a comprehensive behavioral and genetic analysis of a G × E mouse model exhibiting symptoms closely aligned with the pathogenesis of schizophrenia. We found that subchronic administration of PCP induced vulnerability and behavioral abnormalities consistent with the onset of schizophrenia in *Kpna1*-deficient mice. Moreover, in the NAc, G × E interaction-dependent alterations in gene expression (such as dopamine receptors, AMPA receptor editing enzymes, and microtubule motor-related factors) were observed. Our findings indicate that *Kpna1*-deficient mice may be valuable as a G × E interaction mouse model for psychiatric disorders and for further investigation into the pathogenesis of such diseases and disorders.

### Supplementary Information

Supplementary Information 1.

Supplementary Information 2.

Supplementary Information 3.

Supplementary Figures.

## Data Availability

All data generated or analyzed during this study are included in this published article and its supplementary information files. Further information and requests for resources and supporting data will be fulfilled by the corresponding authors.
