# Supplementary material for: A Kpna1-deficient psychotropic drug-induced schizophrenia model mouse for studying gene–environment interactions
Source: Sci Rep. 2024 Feb 9;14:3376. doi: 10.1038/s41598-024-53237-3 (PMC10858057; doi:10.1038/s41598-024-53237-3)
Supplement: Supplementary file 4 — Supplementary Figures. [file 41598_2024_53237_MOESM4_ESM.pdf]

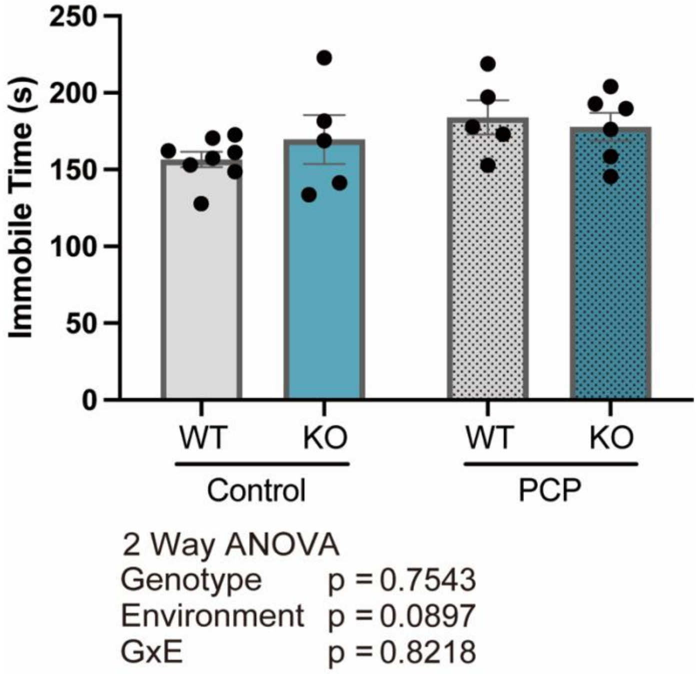

Supplementary Figure S1 Assessment of depression-like behaviors in the FS in control and PCP-treated WT and *Kpna1* KO mice. Time spent immobile (s) in the FS test. The data are presented as the Mean ± SEM. FS, forced swim; PCP, phencyclidine.

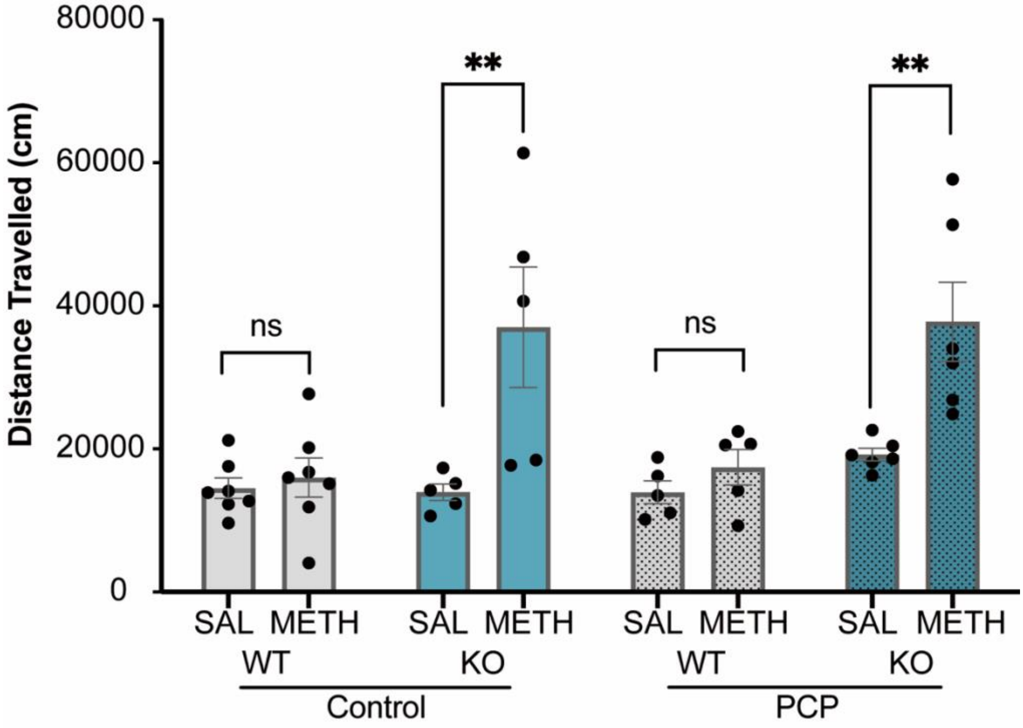

3 Way ANOVA

Genotype	p = 0.0006***
Environment	p = 0.5392
G x E	p = 0.6512
METH	p = 0.0001***
METH x G	p = 0.0014**
METH x E	p = 0.8031
METH x G x E	p = 0.5174

Supplementary Figure S2 Assessment of anxiety-like behaviors in the OFT in control and PCP-treated WT and *Kpna1* KO mice. (a) Distance travelled over 60 min in the OFT with METH or SAL administration. The data are presented as the Mean ± SEM. ** $p < 0.01$, *** $p < 0.001$ Post-hoc Bonferroni Test. OFT, open field test; METH, methamphetamine; SAL, saline; PCP, phencyclidine.

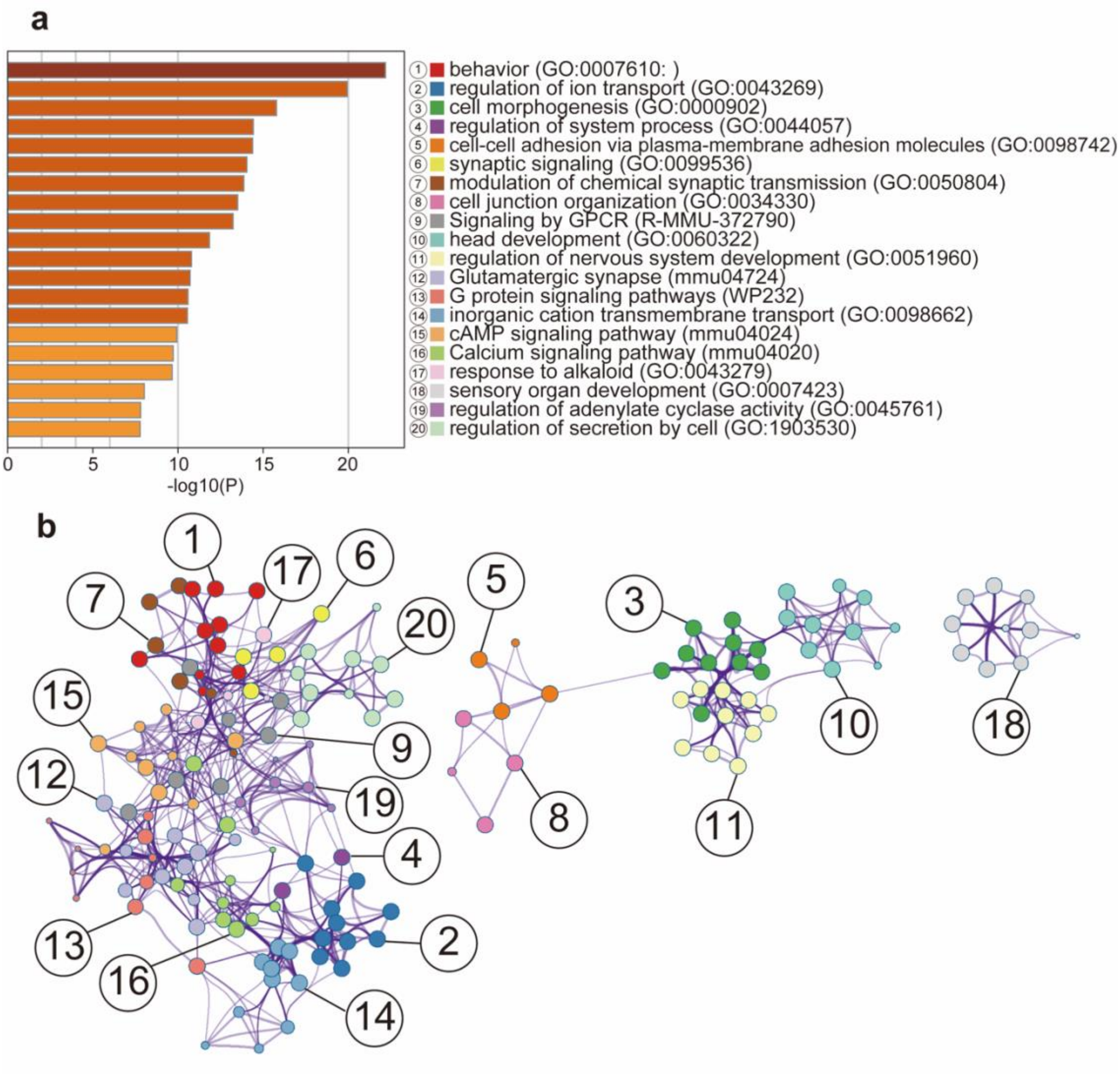

Supplementary Figure S3 GO term enrichment in DEGs upregulated in the NAc. Overrepresented functional terms for DEGs enriched in PCP-treated *Kpna1* KO mice *vs*. PCP-treated WT mice. The nodes of the network represent clusters of GO terms, grouped and colored by comprehensive description. The numbers correspond to the enriched GO terms. The clusters are designated by a number along with a statistical confidence level (log10 p-value) along the x-axis. DEGs, differentially expressed genes; NAc, nuclear accumbens; PCP, phencyclidine; GO, gene ontology.

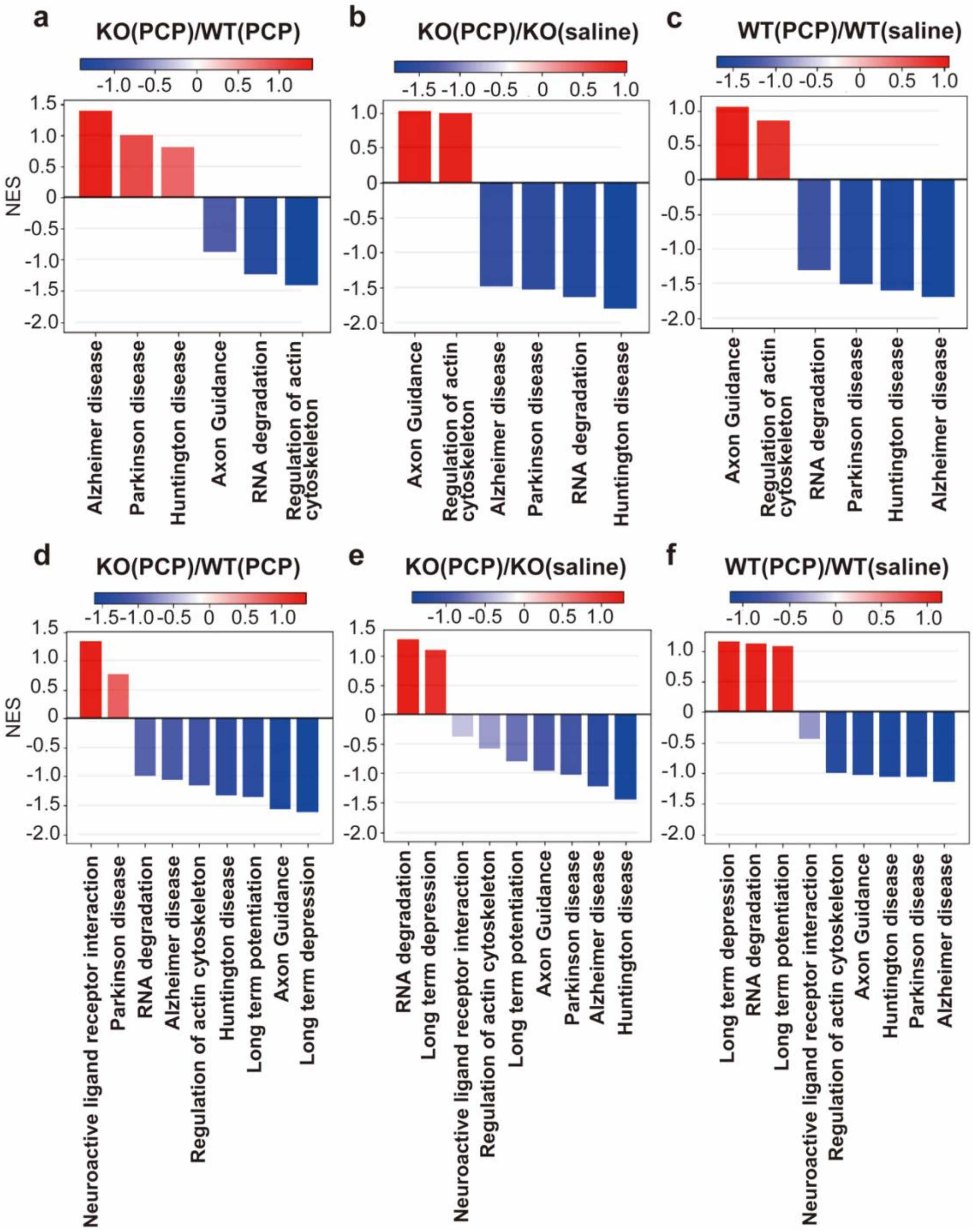

Supplementary Figure S4 Pathway Enrichment in DEGs. Normalized Enrichment Scores (NES) were calculated using the GSEA method. Scale bars are on the far right. (a)-(c) NESs for each pathway in the PFc. (d)-(f) NESs for each pathway in the NAc. DEGs, differentially expressed genes; PFc, prefrontal cortex; NAc, nuclear accumbens; GSEA, gene set enrichment analysis; KO, *Kpna1* KO; PCP, phencyclidine.

(a) PFc

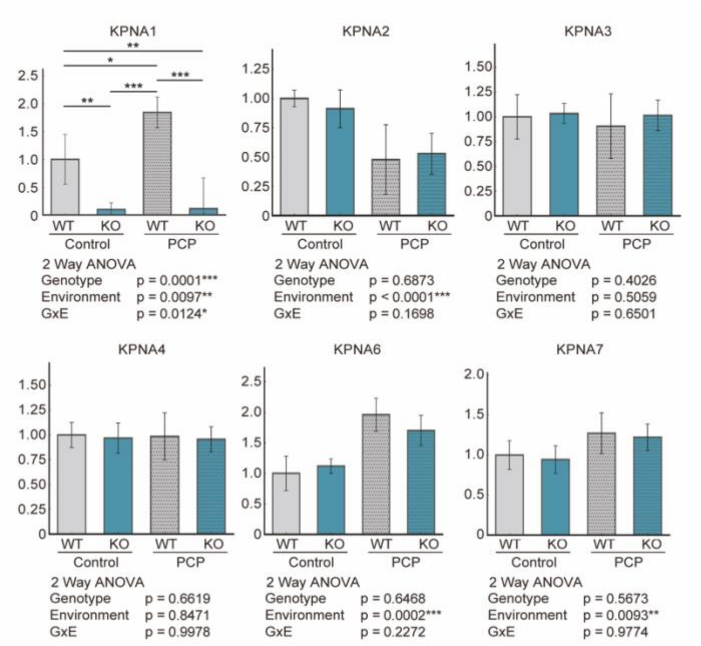

(b) NAc

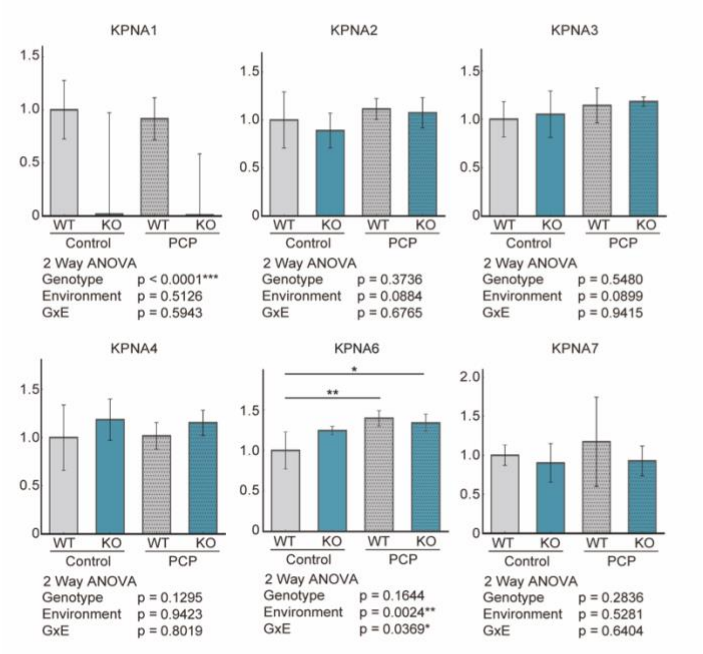

Supplementary Figure S5 Differentially expressed genes. The expression level of WT (saline) is set = 1 and the relative expression level of each KPNA in the (a) PFc and (b) NAc is shown. Two-way ANOVA and post-hoc Tukey test were performed when there was a G x E interaction (p<0.05). * p < 0.05, ** p < 0.01, *** p<0.001 Post-hoc Tukey Test.
